# Influenza and Purulent Bronchitis

**Published:** 1918-11

**Authors:** 


**Influenza and Purulent Bronchitis.** By Capt. A. MAUDE, M. R. C. S., L. R. C. P. Lond. *The Lancet.* September 7, 1918.

The author reports that 2,067 men out of 10,000 were reported sick with influenza in one camp between June 18, and July 18, 1918. A high proportion of these developed purulent bronchitis, while others showed a frank lobar pneumonia, usually of the lower lobes only.

*Physical Signs :* Generally there appeared to be little relation between the extent of physical signs and the rise of temperature.

Usually coarse bronchitis appeared together with lobar pneumonia. In many instances the bronchitis was one-sided, showing its infective quality. Small patches of collapsed lungs were a frequent sequel of this bronchitis; the collapse was of short duration in all cases, and completely cleared up.

*Sputum* : The usual color in the pneumonic cases was a faint crushed strawberry. Eventually the expectoration became yellow and tenacious, consisting of almost pure pus. No bacteriological examination was undertaken, but it seems most likely that double successive infection by the influenza bacillus and the pneumococcus occurred.

*Pleurisy.* Pleuritic patches developed in a large number of cases.

*Complexion.* The "heliotrope" complexion was present in many of the worst cases, but was not always a fatal sign, as even among these many recoveries occurred.

*Temperature.* Temperature rose to 105° or 106° F. in the pneumonic cases; a rapid fall of temperature proved generally delusive and was soon followed by a new rise. When purulent expectoration was well established, a swinging type with a range of several degrees was observed.

*Prognosis.* Three cases only proved fatal, all of extensive double pneumonia.

*Treatment.* Expectorants, combined with free alcoholic stimulation and heart tonics, proved useful. Cold and tepid sponging was resorted to in cases of high temperature. Atropin seemed to interfere with pulmonary secretion and to do more harm than good.

